# Instrument-based tests for quantifying aqueous humour protein levels in uveitis: a systematic review protocol

**DOI:** 10.1186/s13643-019-1206-2

**Published:** 2019-11-26

**Authors:** Thomas W. McNally, Xiaoxuan Liu, Sophie Beese, Pearse A. Keane, David J. Moore, Alastair K. Denniston

**Affiliations:** 1 Academic Unit of Ophthalmology, Institute of Inflammation & Ageing, College of Medical and Dental Sciences, University of Birmingham, Birmingham, UK; 2 Ophthalmology Department, University Hospitals Birmingham NHS Foundation Trust, Birmingham, UK; 3 NIHR Biomedical Research Centre for Ophthalmology, Moorfields Eye Hospital NHS Foundation Trust and UCL Institute of Ophthalmology, Birmingham, UK; 4 Institute of Applied Health Research, College of Medical and Dental Sciences, University of Birmingham, Birmingham, UK; 5 Centre for Rare Diseases, Institute of Translational Medicine, Birmingham Health Partners, Birmingham, UK

**Keywords:** Systematic review, Uveitis, Anterior chamber flare, Intraocular inflammation, Aqueous humour, Proteins, Blood-ocular barrier, Monitoring test, Diagnostic test, Optical coherence tomography, Laser flare photometry, Laser flare meter, Tyndallometry

## Abstract

**Background:**

Inflammation in anterior uveitis is characterised by breakdown of the blood-ocular barrier, which allows leakage of blood constituents of higher molecular weight into the aqueous humour. In routine clinical care, increase in aqueous protein levels can be observed at the slit lamp as ‘flare’ and the severity can be graded using various clinical grading systems, of which the Standardization of Uveitis Nomenclature (SUN) grading system is most commonly used. Alternative instrument-based technologies are available, which can detect aqueous protein levels in an objective and quantifiable way. This review will identify instruments capable of measuring anterior chamber inflammation in this way, their level of reliability, and how well the measurements correlate with clinical grading and/or actual aqueous protein concentration.

**Methods:**

Standard systematic review methodology will be used to identify, select and extract data from studies that report the use of any instrument-based technology in the assessment of aqueous protein levels. Searches will be conducted through bibliographic databases (MEDLINE, EMBASE and Cochrane Library), clinical trial registries and the grey literature. No restrictions will be placed on language or year of publication. The outcomes of interest are the level of correlation between identified instrument-based test measurements, clinical grading and/or actual aqueous protein concentration, as well as the reliability of each index test identified. Study quality assessment will be based on QUADAS2. Correlation and reliability outcomes will be pooled and meta-analysed if appropriate.

**Discussion:**

The assessment of inflammation in anterior chamber protein levels currently relies on crude and subjective clinical examination. The findings of this review will identify non-invasive technologies which show good correlation with actual protein concentration, which could be used in routine clinical practice for objective monitoring of AC inflammation.

**Systematic review registration:**

PROSPERO CRD42017084167. Study screening stage has just been completed.

## Background

Uveitis, a significant cause of blindness worldwide with a global prevalence of 38–114.5 per 100,000 [1, 2], describes a group of conditions characterised by intraocular inflammation [3, 4]. Although uveitis can occur at any age [3], it commonly affects the working-age group and therefore has a substantial socioeconomic impact [5].

Anterior uveitis is characterised by inflammation of the anterior uveal tract. The acute symptoms are primarily pain, redness and photophobia, but vision can be affected. With adequate treatment, these symptoms are reversible; however, permanent and vision-threatening complications (such as posterior synechiae formation, cataract and secondary glaucoma) can develop in the presence of prolonged inflammatory activity.

Inflammation in the anterior chamber (AC) can be observed as changes of the aqueous humour. The aqueous humour is a clear fluid in the anterior chamber of the eye that provides nutrition, removes excretory products of metabolism, transports neurotransmitters, stabilises the ocular structure and contributes to the regulation of the homeostasis of ocular structures [6]. In health, the aqueous humour is well isolated from the blood by blood-aqueous barriers including the endothelial cells of the iris capillaries and retinal vessels and non-pigmented ciliary body epithelium. The aqueous is mostly water, but also contains electrolytes, carbohydrates, glutathione, urea, oxygen, carbon dioxide, and proteins [6, 7]. In the absence of inflammation, only small proteins are present in low concentrations in the aqueous. Inflammation causes breakdown of the blood-aqueous barriers and results in the release of inflammatory cells and proteins of higher molecular weight into the eye [8, 9]. The accumulation of proteins in the aqueous humour can be observed as flare or Tyndall effect, which is an optical phenomenon of cloudiness in the aqueous humour due to increased protein content.

The ability to accurately measure the concentration of proteins in the aqueous is clinically important for detecting acute inflammatory episodes and assessing response to treatment [10]. There are several established methods for quantifying aqueous protein levels, including clinical examination and laser flare photometry. However, with the exception of aqueous paracentesis (invasive sampling of the aqueous humour), existing methods are surrogate measures relating to the change in optical properties of the aqueous humour when protein levels are increased. Each method has its limitations, and it is unclear whether measurements from different methods agree with each other. The following section discusses the pros and cons of existing measures for aqueous protein concentration.

### Invasive quantification of aqueous protein sampling: aqueous paracentesis

Currently, the only means of directly measuring the protein content of the aqueous is by aqueous paracentesis and analysis in a laboratory setting [11, 12]. While this method provides the most accurate quantification of aqueous constituents, it is not used routinely in clinical practice as it is invasive and carries risk of sight-threatening complications [13]. Consequently, its use is mainly limited to the research setting, and non-invasive methods are used preferably in clinical practice.

### Non-invasive quantification of aqueous protein level

#### Clinical examination

The current clinical standard for AC flare grading was defined at the Standardisation of Uveitis Nomenclature (SUN) working group consensus meeting in 2005 [4]. Prior to this, several systems existed for grading AC flare [14–17]. Slit-lamp biomicroscopy enables a clinician to grade increasing protein concentrations in the aqueous humour due to back-scattering of light emitted from a slit-lamp. The SUN system grades flare according to the observer’s ability to visualise details of the iris and lens behind the aqueous. Grades range from 0, which corresponds to no visible flare, to 4+, which corresponds to intense flare (Table 1).

**Table 1:** The Standardisation of Uveitis Nomenclature (SUN) working group grading scheme for anterior chamber flare [4]

Grade	Flare	Description
0	None	
1+	Faint	Barely detectable
2+	Moderate	Iris and lens details clear
3+	Marked	Iris and lens details hazy
4+	Intense	Fixed coagulated aqueous with considerable fibrin

Source: Ref [4]

Grading of flare based on clinical examination is subjective and subject to high intra- and inter-observer variability [18]. In addition, it is a non-continuous scale, leading to large steps in disease activity between categories. Detection and monitoring of inflammatory changes in anterior uveitis is critically important for clinical management, decision making and clinical trials investigating therapeutic agents [19]. The need for a more robust and reproducible method of measurement is well-recognised within the uveitis community. Newer imaging techniques have become available in recent years; therefore, it is timely to carry out a review of whether they can be purposed for this unmet need and evaluate how they compare to the current standard.

#### Laser flare and cell photometry

In 1988, Sawa et al. first described laser flare photometry (LFP) as a new method to precisely determine protein concentration in the aqueous humour [20]. LFP is a rapid and non-invasive technique which quantifies AC flare by projecting a laser beam through the anterior chamber and measuring the amount of back-scattered light. Particles present in the aqueous reflect photons of light, which are then measured by an inbuilt photomultiplier. The intensity of back-scattered light is proportional to the concentration and size of proteins in the aqueous chamber. Most models of LFP are capable of measuring flare, but the technology can also be used to count AC cells. However, assessment of AC cells using LFP is less accurate as inflammatory cells cannot be differentiated from other large particles such as pigment particles, debris or red blood cells [9].

#### Optical coherence tomography (OCT)

OCT is an imaging technique which has become available more recently. It uses coherent light to capture high resolution 2- and 3-dimensional images of structures within the eye. It is a fast and non-invasive method of obtaining high precision images and measurements of ocular structure [21]. Anterior segment OCT (AS-OCT) can provide cross-sectional imaging of the AC. Recently, OCT has demonstrated the ability to detect higher light reflectivity signals on AS-OCT during active inflammation [21].

### Aim

The primary aim of this review is to identify all available non-invasive, instrument-based techniques with the potential to quantify protein levels in the aqueous as a measure of AC inflammation. We will evaluate the level of correlation of these measurements with clinical grading using slit lamp examination and/or actual aqueous protein concentration. Where reported, we will also evaluate the level of reliability of each test.

A secondary aim of the review is to evaluate the correlation between clinical grading and protein concentration measured by laboratory analysis of aqueous samples.

The following questions are proposed:

- Which index tests have the potential to quantify AC inflammation in uveitis?
  - What is the level of correlation between the index test and clinical grading?
  - What is the level of correlation between the index test and aqueous protein concentration?
  - What is the level of repeatability of each test?
- What is the level of correlation between clinical grading using the slit lamp and aqueous protein concentration?

## Methods

### Protocol

This protocol has been designed in accordance with the guidelines of Preferred Reporting Items for Systematic Review and Meta-Analysis Protocol (PRISMA-P) [22]. The systematic review will be reported in accordance to the PRISMA guidelines [23].

### Systematic review registration

This systematic review has been registered on PROSPERO (CRD42017084167) [24].

### Searches

#### Databases

Our search strategy will include the following areas of interest: anatomical location (‘anterior chamber’), the pathological finding (‘flare’) and the disease context (‘uveitis’). To achieve optimal sensitivity, no search terms will be applied for the ‘technologies/tests’. For bibliographic databases, free text and index terms will be combined for each search element where possible. A sample search strategy for MEDLINE is included in the Appendix 1. We will search:

- Bibliographic databases of published studies.
  - MEDLINE (Ovid), 1946 to present
  - Embase (Ovid), 1947 to present
  - Cochrane Central Register of Controlled Trials (CENTRAL), database inception to present
  - Centre for Reviews and Dissemination database (Health Technology Assessments and the Database of Abstracts and Reviews of Effects), database inception to present
- Registers of clinical trials
  - Clinicaltrials.gov. www.clinicaltrials.gov
  - WHO International Clinical Trials Registry Platform (ICTRP portal). www.who.int/ictrp.
- Abstract and conference proceedings
  - British Library’s ZETOC.
  - Conference proceedings Citation Index (Web of Science).
- Dissertations, theses
- British library Ethos
- ProQuest. www.proquest.com
- Grey literature
  - OpenGrey. www.opengrey.eu

No restrictions will be placed on year or language of publication. The literature search results will be entered onto EndNote (Clarivate Analytics, Philadelphia, PA) to facilitate removal of duplicates, study selection, recording decisions and references. References to other works will be considered for inclusion.

### Selection criteria

#### Participants/population

Those with evidence of anterior chamber flare and/or a diagnosis of uveitis, irrespective of active or inactive inflammation, will be included. There will be no restrictions on age, gender, ethnicity, or underlying aetiology or anatomical subtype. Studies with only healthy participants and studies on animals will not be included.

#### Index test

Any study reporting one or more non-invasive, instrument-based technology with the potential for quantifying AC flare or aqueous protein measurements will be included. To address the secondary outcome (clinical grading versus laboratory measurements of aqueous proteins), any clinical grading system can be considered the index test.

#### Reference test

The reference test should be either the current clinical standard—clinician-based AC flare grading, or the laboratory standard—measurements of aqueous protein from aqueous paracentesis.

For the clinical grading versus laboratory analysis, the reference test must be a laboratory-based technique for measuring aqueous protein concentration of extracted aqueous samples.

#### Primary outcomes

The level of correlation (correlation coefficient) of index test measurements with clinical grading using slit lamp examination and/or actual aqueous protein concentration.

#### Secondary outcome

Intra/inter-observer reliability and repeatability of an index test (kappa statistic) and the level of correlation between clinical grading and actual protein concentration (clinical grading as the index test and protein concentration as the reference test).

#### Type of study

There will be no restrictions on study design; however, evaluation of correlation between index test and clinical grading using slit-lamp examination/aqueous protein measurements require both tests to be done in a cross-sectional manner. If multiple time points are included, we will include results from all time points, but comparisons between tests will be cross-sectionally. Only those studies where measurements are taken within a reasonable time point (within 24 h of each other) will be included. Case reports involving only one subject, commentaries, opinion articles and pictorial articles will not be included.

### Selection process

Titles and abstracts of studies will be screened for relevance to the review, to remove obviously irrelevant studies Two independent reviewers will carry out study selection and reach consensus by discussion or referral to a third reviewer.

Full text of potentially relevant articles will be retrieved and assessed for inclusion in the review against the full selection criteria.

### Data extraction

A standardised data extraction form in Microsoft Excel (Microsoft, Washington, US) will be used to extract data from included studies. The extraction process will be carried out by two independent reviewers with referral to a third reviewer if necessary. Information extracted from all studies will include:

- Study characteristics
  - Title, authors, publication year, journal and language
  - Sample size
  - Study design
  - Index test used
    1. Manufacturer and model (including resolution, default settings)
    2. Measurements acquisition protocol
    3. Measurement analysis protocol
  - Clinical grading system measurements
    1. Clinical grading system used and whether any modifications were made
    2. Number of observers
  - Aqueous protein concentration
    1. Context and reason for aqueous extraction
    2. Aqueous extraction protocol
    3. Aqueous analysis protocol
- Patients’ characteristics
  - Age, gender and ethnicity
  - Underlying aetiologies (anatomical subtype, aetiological classification)
  - Active or inactive disease
  - If the study involves a therapeutic intervention: treatment details (indication, drug, dosage, route, subject pre/post treatment status, length of follow-up and number of time points suitable for review question)
- Outcomes and findings
  - Data will be extracted in preparation for four separate analyses:
    1. Evaluation of correlation between index tests and actual aqueous protein concentration: The correlation coefficient will be directly extracted.
    2. Evaluation of correlation between index tests and clinical grading systems: The correlation coefficient reported will be directly extracted.
    3. Evaluation of correlation between the clinical grading system and aqueous protein concentration: The correlation coefficient will be directly extracted.
    If no correlation coefficient is reported, Index and reference test measurements will be extracted and, provided they are matched, used to calculate the correlation coefficient. If the two measurements are not matched, we will contact the authors for matched measurements.
    4. Evaluation of reliability and repeatability of an index test: Studies reporting intra- and inter-observer reliability will be analysed separately for assessment of repeatability. The reported kappa values will be extracted for intra-observer reliability, inter-observer reliability or both.

Cross-sectional measurements may be nested within longitudinal studies whose aims are not primarily to compare performance or correlation between two tests. In this situation, measurements at each time point will be extracted and analysed as individual cross-sectional comparisons.

### Quality assessment

All included studies will be assessed for quality using elements of the Quality Assessment of Diagnostic Accuracy Studies tool (QUADAS2) [25]. Although QUADAS2 is designed for comparison of diagnostic accuracy, rather than agreement of test measurements, it is the most suitable existing tool for evaluating risk of bias in diagnostic tests. We have modified elements of the existing QUADAS2 signalling questions to suit the aims of this systematic review (see Appendix 2). For example, the original QUADAS2 framework includes a question regarding whether thresholds were used and pre-specified. We have removed this item as we are not aiming to assess diagnostic accuracy. Similarly, we have supported the QUADAS2 with additional items such as the addition of the question ‘were index test acquisition and analysis parameters determined a priori and consistent for all study participants?’ Existing questions regarding blinding during test interpretation, applicability to the review question, and flow and timing of tests are kept as they remain important for this review.

Assessment will be carried out at the study level. Two independent reviewers will carry out quality assessment and refer to a third reviewer if needed. For studies assessing correlation between two tests, the four risks of bias domains below will be rated as low, high or unclear.

For studies investigating the reliability of a single index test, additional considerations will be made for:

1. Intra-observer reliability studies: the conditions under which the index test was performed should be reported and standardised.
2. Inter-observer reliability studies: any differences between observer characteristics, such as seniority and experience, should be reported.

### Data synthesis

Studies will be included in four groups for data synthesis, one for each outcome: correlation between index test and the clinical grading, correlation between index test and aqueous protein concentration, correlation between the SUN grading system and laboratory measurement of aqueous protein concentration, and reliability of index test.

For each outcome, a narrative synthesis of tabulated evidence will be conducted and, where possible, supported with a meta-analysis.

1. Evaluation of correlation between index test and the SUN grading system
  These studies will be grouped by the type of technology used for the index test (i.e. LFP, OCT). If the data permits, correlation coefficients between each index test versus clinical grade will be compared and pooled for meta-analysis using a random effects model.
2. Evaluation of correlation between index test and laboratory measurement of aqueous protein concentration
3. Evaluation of correlation between the SUN grading system and laboratory measurement of aqueous protein concentration
4. Evaluation of reliability of an index test
  These studies will be grouped by type of technology as above. We will analyse intra- and inter-observer reliability separately for each technology. If the data permits, reliability values (such as kappa values) will be compared and pooled for meta-analysis.

Studies will be assessed for clinical and methodological homogeneity to determine whether it is possible appropriately pool data for meta-analysis. The *I*^2^ statistic will be used to quantify heterogeneity across studies in each meta-analysis. Correlation coefficients will be normalised using Fisher’s *Z* transformation for meta-analysis and back transformed for inference. Meta-analysis of kappa statistics will be performed using an inverse-variance weighted random effects model with standard errors estimated from reported 95% confidence intervals. All data will be reported narratively, regardless of whether data pooling and meta-analysis are possible or not.

Depending on the study data, appropriate sensitivity analyses will be carried out if there is significant heterogeneity between studies. Subgroup analyses for anatomical and aetiological subtypes of uveitis, index test technology and experience of graders (for clinical AC flare grading) will be considered to explore sources of heterogeneity. It is expected that only a small number of studies will meet inclusion criteria, which will limit our capacity to carry out meaningful subgroup analyses.

## Discussion and potential impact

The assessment of inflammation in anterior uveitis currently relies on imperfect clinical methods. An increase in aqueous protein concentration is a detectable change which occurs in the presence of inflammatory breakdown of the blood ocular barrier. Laboratory measurement of aqueous protein concentration in aqueous samples obtained through paracentesis is a good gold standard test but carries too many risks to make it practical for monitoring patients. Thus, clinicians must resort to surrogate markers for aqueous protein levels such as ‘flare’.

Whilst the SUN grading system marked a significant effort towards unifying the method for assessing uveitic inflammation, it continues to rely on clinical examination and the subjective appearance of aqueous ‘clarity’. Many factors can also affect an observer’s ability to observe flare including the slit-lamp optics, degree of illumination, ambient conditions and the observer’s level of expertise [26].

The potential for instrument-based techniques for assessing AC inflammation carries significant advantages over clinical grading systems because they are objective, less operator dependent and produce a continuous numerical value that is precise at even low levels of inflammation. Objective measures of inflammation for monitoring AC cells and vitreous haze have been proposed, and OCT measurement of central macular thickness is already established in routine clinical practice [19, 21, 27, 28]. With the use of automated analysis software, these techniques could allow comprehensive objective disease monitoring, potentially even in a virtual care model.

To date, the use of non-invasive instrument-based techniques for assessing AC flare has not been assessed in a systematic way. The need for more objective measures of disease activity is well-recognised within the uveitis community, and given the rise in new imaging technologies over recent years which have the potential to meet this demand, it is timely to carry out a review of how they compare to the current standard. This systematic review will identify all technologies available for quantifying ‘flare’ and their level of reliability and correlation with laboratory measurement of aqueous protein concentration and/or clinical grading. The findings of this review could contribute to the validation process of instrument-based methods for monitoring inflammatory activity and guiding treatment decisions in uveitis.

## Data Availability

Data sharing is not applicable to this article as no datasets were generated or analysed during the current study.

## Appendix 1

**Table 2:** MEDLINE search strategy

Number	Search
1	Anterior Chamber. Ti,ab.
2	Aqueous. Ti,ab.
3	Anterior segment. Ti,ab.
4	1 or 2 or 3
5	Flare*. Ti,ab,
6	Photon. Ti,ab.
7	Photons. Ti,ab.
8	Protein. Ti,ab.
9	Proteins. Ti,ab.
10	Photometry. Ti,ab.
11	Fluorophotometry. Ti,ab.
12	Tyndal* Ti,ab.
13	5 or 6 or 7 or 8 or 9 or 10 or 11 or 12
14	Exp Uveitis/
15	Uveiti*. Ti,ab.
16	Inflamm*. Ti,ab.
17	Blood aqueous barrier. Ti,ab.
18	14 or 15 or 16 or 17
19	4 and 13 and 18

## Appendix 2

**Table 3:** Modified elements of the QUADAS2 signalling questions used for quality assessment

Domain	Patient selection	Index test*	Reference standard	Flow and timing
Description	Describe methods of patient selection: Describe included patients (prior testing, presentation, intended use of index test and setting):	Describe the index test and how it was conducted and interpreted:	Describe the reference standard and how it was conducted and interpreted:	Describe any patients who did not receive the index test(s) and/or reference standard or who were excluded from the 2x2 table (refer to flow diagram): Describe the time interval and any interventions between index test(s) and reference standard:
Signalling questions (yes/no/unclear)	Was a consecutive or random sample of patients enrolled?	Were the index test results interpreted without knowledge of the results of the reference standard?	Is the reference standard likely to correctly classify the target condition?	Was there an appropriate interval between index test(s) and reference standard?
	Was a case-control design avoided?	*Were index test acquisition and analysis parameters determined a priori and consistent for all study participants?*	Were the reference standard results interpreted without knowledge of the results of the index test?	Did all patients receive a reference standard?
	Did the study avoid inappropriate exclusions? *i.e. Participants may be excluded if justified in terms of interference with index test measurement (corneal opacities preventing visualisation of anterior structures).*			Did all patients receive the same reference standard? *i.e. Was the reference test conducted in the same way for each patient?* *For slit lamp examination: observer, slit lamp settings* *For laboratory protein measurements: method of aqueous extraction, sample storage and analysis.*
				Were all patients included in the analysis?
Risk of bias: High/low/unclear	Could the selection of patients have introduced bias?	Could the conduct or interpretation of the index test have introduced bias?	Could the reference standard, its conduct, or its interpretation have introduced bias?	Could the patient flow have introduced bias?
Concerns regarding applicability: High/low/unclear	Are there concerns that the included patients do not match the review question?	Are there concerns that the index test, its conduct, or interpretation differ from the review question?	Are there concerns that the target condition as defined by the reference standard does not match the review question?	

Italics denote signalling questions added by the authors for this systematic review.

*In index test, the signalling question regarding whether thresholds were used and pre-specified is not applicable for this review and was therefore removed

## Abbreviations

AC: Anterior chamber
AS-OCT: Anterior segment OCT
LFP: Laser flare photometry
MEDLINE: Medical Literature Analysis and Retrieval System Online
OCT: Optical coherence tomography
PRISMA: Preferred Reporting Items for Systematic Review and Meta-Analysis
PRISMA-P: Preferred Reporting Items for Systematic Review and Meta-Analysis Protocol
QUADAS 2: Quality Assessment of Diagnostic Accuracy Studies
SUN: Standardisation of Uveitis Nomenclature

## References

[1] Williams GJ, Brannan S, Forrester JV, Gavin MP, Paterson-Brown SP, Purdie AT (2007). The prevalence of sight-threatening uveitis in Scotland. Br J Ophthalmol..

[2] Rothova A, Suttorp-van Schulten MS, Frits Treffers W, Kijlstra A (1996). Causes and frequency of blindness in patients with intraocular inflammatory disease. Br J Ophthalmol..

[3] Durrani OM, Meads CA, Murray PI (2004). Uveitis: a potentially blinding disease. Ophthalmologica..

[4] Jabs DA, Nussenblatt RB, Rosenbaum JT; Standardization of Uveitis Nomenclature (SUN) Working Group. Standardization of uveitis nomenclature for reporting clinical data. Results of the First International Workshop. Am J Ophthalmol. 2005;140:509–16. 10.1016/j.ajo.2005.03.057 PMC8935739 16196117

[5] Lardenoye CWTA, van Kooij B, Rothova A (2006). Impact of macular edema on visual acuity in uveitis. Ophthalmology..

[6] Goel M, Picciani RG, Lee RK, Bhattacharya SK (2010). Aqueous humor dynamics: a review. Open Ophthalmol J..

[7] Murthy Krishna R., Rajagopalan Pavithra, Pinto Sneha M., Advani Jayshree, Murthy Praveen R., Goel Renu, Subbannayya Yashwanth, Balakrishnan Lavanya, Dash Mahashweta, Anil Abhijith K., Manda Srikanth S., Nirujogi Raja Sekhar, Kelkar Dhanashree S., Sathe Gajanan J., Dey Gourav, Chatterjee Aditi, Gowda Harsha, Chakravarti Shukti, Shankar Subramanian, Sahasrabuddhe Nandini A., Nair Bipin, Somani Babu Lal, Prasad T. S. Keshava, Pandey Akhilesh (2015). Proteomics of Human Aqueous Humor. OMICS: A Journal of Integrative Biology.

[8] Shah SM, Spalton DJ, Taylor JC. Correlations between laser flare measurements and anterior chamber protein concentrations. Investig Ophthalmol Vis Sci. 1992;33:2878–84. 1526738

[9] Tugal-Tutkun I, Herbort CP (2010). Laser flare photometry: a noninvasive, objective, and quantitative method to measure intraocular inflammation. Int Ophthalmol..

[10] Tugal-Tutkun I, Cingu K, Kir N, Yeniad B, Urgancioglu M, Gul A (2008). Use of laser flare-cell photometry to quantify intraocular inflammation in patients with Behcet uveitis. Graefes Arch Clin Exp Ophthalmol..

[11] Saari K. M., Guillén-Monterrubio O. M., Hartikainen J., Hämäläinen M. M., Taskinen K. (2009). Measurement of protein concentration of aqueous humour in vivo: correlation between laser flare measurements and chemical protein determination. Acta Ophthalmologica Scandinavica.

[12] Davis JL, Ruiz P Jr, Shah M, Mandelcorn ED. Evaluation of the reactive T-cell infiltrate in uveitis and intraocular lymphoma with flow cytometry of vitreous fluid (an American Ophthalmological Society thesis). Trans Am Ophthalmol Soc. 2012;110:117–29. PMC3671364 23818738

[13] Cheung CMG, Durrani OM, Murray PI (2004). The safety of anterior chamber paracentesis in patients with uveitis. Br J Ophthalmol..

[14] Nussenblatt RB, Whitcup SM (2004). Uveitis: fundamentals and clinical practice 3rd.

[15] Hogan Michael J., Kimura Samuel J., Thygeson Phillips (1959). Signs and Symptoms of Uveitis*. American Journal of Ophthalmology.

[16] Schlaegel T (1967). Essentials of uveitis.

[17] Foster CS, Vitale AT. Diagnosis and treatment of uveitis. Philadelphia: WB Saunders; 2002.

[18] Kempen JH, Ganesh SK, Sangwan VS, Rathinam SR (2008). Interobserver agreement in grading activity and site of inflammation in eyes of patients with uveitis. Am J Ophthalmol..

[19] Denniston Alastair K., Keane Pearse A., Srivastava Sunil K. (2017). Biomarkers and Surrogate Endpoints in Uveitis: The Impact of Quantitative Imaging. Investigative Opthalmology & Visual Science.

[20] Sawa M, Tsurimaki Y, Tsuru T, Shimizu H (1988). New quantitative method to determine protein concentration and cell number in aqueous in vivo. Jpn J Ophthalmol..

[21] Invernizzi A, Marchi S, Aldigeri R, Mastrofilippo V, Viscogliosi F, Soldani A (2017). Objective quantification of anterior chamber inflammation: measuring cells and flare by anterior segment optical coherence tomography. Ophthalmology..

[22] Moher D, Shamseer L, Clarke M, Ghersi D, Liberati A, Petticrew M, et al. Preferred reporting items for systematic review and meta-analysis protocols (PRISMA-P) 2015 statement. Syst Rev. 2015;4:1. 10.1186/2046-4053-4-1 PMC4320440 25554246

[23] Moher D, Liberati A, Tetzlaff J, Altman DG (2010). PRISMA Group. Preferred reporting items for systematic reviews and meta-analyses: The PRISMA statement. Int J Surg..

[24] Liu X, McNally T, Beese S, Keane P, Moore D, Denniston AK. PROSPERO Instrument-based tests for measuring anterior chamber (AC) flare in uveitis: a systematic review. PROSPERO. 2018. https://www.crd.york.ac.uk/prospero/display_record.php?RecordID=84167.

[25] Whiting Penny F. (2011). QUADAS-2: A Revised Tool for the Quality Assessment of Diagnostic Accuracy Studies. Annals of Internal Medicine.

[26] Wong IG, Nugent AK, Vargas-Martín F (2009). The effect of biomicroscope illumination system on grading anterior chamber inflammation. Am J Ophthalmol.

[27] Sharma S, Lowder CY, Vasanji A, Baynes K, Kaiser PK, Srivastava SK (2015). Automated analysis of anterior chamber inflammation by spectral-domain optical coherence tomography. Ophthalmology..

[28] Montesano G, Way CM, Ometto G, Ibrahim H, Jones PR, Carmichael R (2018). Optimizing OCT acquisition parameters for assessments of vitreous haze for application in uveitis. Sci Rep..

